# Evaluating the Efficacy of Combined Platelet‐Rich Plasma and Microneedling for Aesthetic Rejuvenation of the Periorbital Area: A Randomized, Blinded Cohort Study

**DOI:** 10.1111/jocd.16717

**Published:** 2024-12-08

**Authors:** Sally Al Hassan, Dana S. Saade, Mazen Kurban, Jihane Abou Rahal, Ramzi M. Alameddine

**Affiliations:** ^1^ American University of Beirut Medical Center Beirut Lebanon

**Keywords:** aesthetic rejuvenation, microneedling, periorbital area, platelet‐rich plasma, randomized study

## Abstract

**Introduction:**

Microneedling, a technique involving controlled dermal microwounding, and platelet‐rich plasma (PRP) injections are both employed for skin rejuvenation. While both treatments individually show promise, limited research has explored their combined efficacy. This study aimed to evaluate the effectiveness of combining PRP injections with microneedling for aesthetic concerns around the eyes under standardized conditions.

**Methods:**

This single‐center, longitudinal cohort study was conducted from October 2017 to October 2020. Thirteen adult participants (mean age 35, 92.3% female) with aesthetic concerns in the periorbital area underwent combined PRP and microneedling treatment. Standardized photographs were taken preprocedure, at 1 week, and 3 months. Photographs were evaluated by three blinded dermatologists using validated photonumeric scales. Participants completed a self‐assessment questionnaire at the 1‐week follow‐up regarding skin homogeneity, texture, pigmentation, and wrinkles.

**Results:**

Self‐reported outcomes indicated perceived improvements in skin homogeneity (72.7% reporting mild or significant improvement) and texture (81.8% reporting mild, moderate, or significant improvement). Improvements in pigmentation and wrinkles were less pronounced. Dermatologists' assessments revealed no significant differences in skin attributes before and after treatment.

**Conclusion:**

Despite subjective reports of improvement, objective evaluations by independent dermatologists did not demonstrate significant changes. Variability in outcomes might be attributed to differences in study design, treatment protocols, and assessment methods. Further research with larger sample sizes and multiple treatment sessions is needed to better understand the benefits of combining PRP with microneedling for periorbital rejuvenation.

## 1 Introduction

Microneedling is the technique that involves the use of multiple needles at controlled depths to create thousands of microwounds in the dermis that stimulates the dermal wound healing cascade [1, 2]. Like other ablative treatments, this controlled damage results in the stimulation of the body's regenerative mechanisms to improve the skin quality [1].

Platelet‐rich plasma (PRP) injections are thought to contribute to skin rejuvenation through fibroblast activation, collagen synthesis, and by providing other matrix components and various growth factors [3]. Although there have been multiple papers looking at the individual effect of PRP or microneedling for aesthetic concerns, there has been very few studies looking into the efficacy of their combined use.

Our study aimed to evaluate the effectiveness of combining PRP injections with microneedling for aesthetic concerns around the eyes. Given the scarcity of previous reports in the periocular area, and the large variety in reported preparation methods, and different criteria of treatment outcomes, this study offers an objective evaluation of their combined efficacy under standardized conditions and randomized blinded dermatologists.

## 2 Methods

### 2.1 Study Design

This is a single‐center longitudinal cohort study involving patients who underwent PRP injections combined with microneedling treatment (PRP‐microneedling) specifically targeting the eyelid and periorbital area standardized facial photographs were captured at each visit, both before and after the procedure. These images were then evaluated by dermatologists using a fully blinded randomization process, ensuring that the sequence of the photographs was completely concealed. Additionally, patients completed a postprocedure satisfaction questionnaire. All participants, including both physicians and patients, provided written informed consent. The study protocol received approval from the Institutional review board (IRB, BIO‐2017‐0427). This research was conducted at an academic tertiary care center clinic from October 2017 to October 2020.

### 2.2 Participants

Eligible participants for this study were adults aged between 18 and 70 who presented at the clinic with aesthetic concerns related to the under‐eye area, such as dark circles, rhytids, or poor skin quality, and expressed an interest in undergoing a combined PRP‐microneedling treatment. Exclusion criteria encompassed pregnant or lactating individuals, those with blood or platelet disorders, a personal or family history of genetic collagen disease, a history of herpetic disease or other skin problems, and a history of eyelid or cosmetic surgery. Additionally, patients who had undergone any aesthetic treatments, including botulinum toxin within the past 6 months, dermal fillers at any time in the past, or any mechanical or energy‐based procedures such as laser or chemical peel within the past 2 years, were excluded from the study. Participants were instructed to abstain from using therapeutic skincare substances or undergoing any additional cosmetic procedures throughout the study duration.

### 2.3 Intervention

At the first treatment session, the medical history and allergies were reviewed, and patients signed an informed consent. Topical anesthetic cream (EMLA, AstraZeneca, London, UK) was applied to the face approximately 30 min before the procedure. Peripheral venous blood was obtained through venipuncture of peripheral veins from the upper extremity. Around 30 mL of venous blood was collected and placed in acid citrate dextrose (ACD) tubes (BD, Franklin Lakes, USA). PRP was then prepared using the “PRP method” that involves a two‐step centrifugation process [4]. The first centrifugation was run at a parameter of 900 g for 5 min, and the supernatant plasma and buffy coat were then transferred to empty tubes and run for a second spin at 1000 g for 10 min. The collected PRP was then mixed with calcium citrate for platelet activation at a ratio of 0.9 mL PRP with 0.1 mL calcium citrate. Microneedling was done using a derma pen with 36‐needle tips, set at a depth of 1.5 mm, and applied in series in two consecutive passes on the eyelid and the periorbital area including crow's feet, cheeks, zygoma, and temples. PRP was topically applied to the treated area during and after the treatment, and then 0.1 mL aliquots of PRP were injected in the eyelid and periorbital area at the mid‐dermis level using a 30‐gauge needle in serial punctures 1 cm apart. Patients were instructed to leave the topically applied PRP for at least 30 min before cleaning. For wound care, patients were asked to use petroleum‐based emollients (Aquaphor Healing Ointment; Beiersdorf) for 7 days following the procedure and avoid using chemicals and sun exposure.

### 2.4 Photography

**FIGURE 1:**
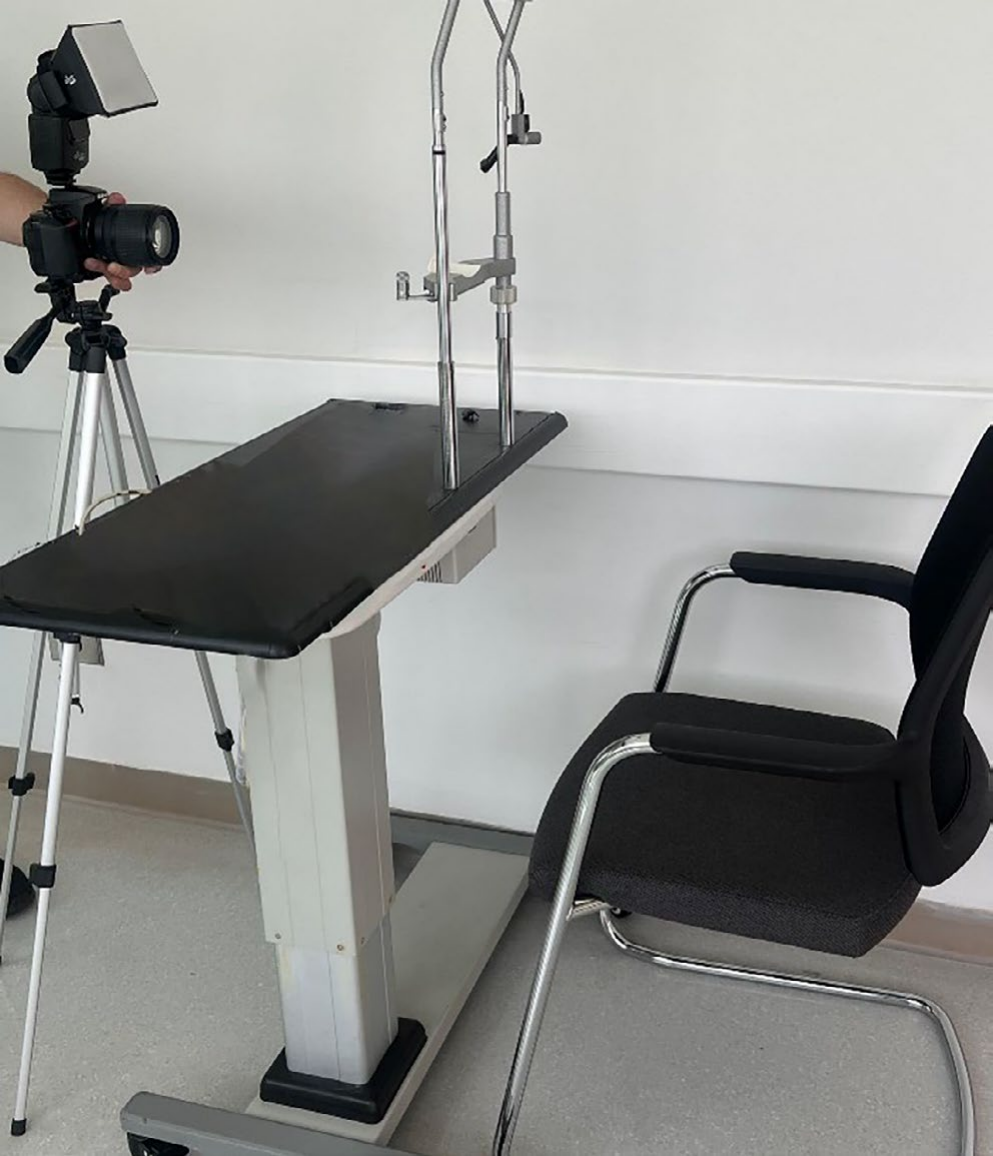
Standardized photography setup. This figure illustrates the standardized setup, including lighting, positioning, and shadow control, used for seating patients during their photosessions.

Standardized digital face photographs were captured during the preprocedure visit, as well as at the 1‐week and 3‐month follow‐up visits. Participants were instructed to remove any makeup and comfortably sit on an adjustable‐height table equipped with fixed head and chin rest fixtures (refer to Figure 1). A digital single‐lens reflex camera (Nikon D5500, Nikon, Japan) equipped with an 85‐mm focal length lens and a light softbox diffuser was securely mounted on a tripod, adjusted to the patient's eye level. All photographs were taken in a consistent, windowless room under uniform lighting conditions, utilizing the same manually set camera settings (F9, ISO 1600, 1/20, 75° flash diffuser, Figure 1).

### 2.5 Outcomes

Participants were asked to complete a self‐assessment questionnaire 1 week following the procedure. The questionnaire included the patient's own assessment of changes in skin homogeneity, texture, pigmentation, and wrinkles each on a four‐point scale (0 = no, 1 = mild, 2 = moderate, 3 = significant improvement). Pain during and after the procedure as well as downtime and any adverse events were also noted.

Furthermore, the standardized face photographs of all participants were anonymized to identifiers such as date taken, and their sequence order was randomized using an online randomizer application, and then digitally shared with three independent dermatologists for grading.

The three blinded dermatologists (D.S., M.K., and J.A.) graded each patient's photographs by using validated photonumeric scales for skin texture, infraorbital pigmentation, fine lines, and crow's feet [5, 6, 7, 8, 9]. Each of the scales was composed of a 5‐point severity score and was guided with verbal and photographic standardized descriptors for each grade (0 = none, 1 = mild, 2 = moderate, 3 = severe, and 4 = extreme or diffuse).

### 2.6 Statistical Analysis

Photonumeric scores and participants' self‐satisfaction questionnaires were analyzed using SPSS. Photonumeric scores were analyzed using a nonparametric *K*‐related samples test, statistical significance threshold was 2‐sided (*p* = 0.05). The agreement between the three dermatologists was tested using the Interclass correlation coefficient. The average measure was used as we compared the averages of the three dermatologists.

## 3 Results

A total of 13 patients were recruited to participate in the study. The majority were females (92.3%) with a mean age of 35 years (range 22–57). During the PRP‐microneedling procedure, most participants reported no pain (45.5%) or mild pain (45.5%). In the first few hours following the procedure, most participants reported no pain (81.8%), and only 2 patients (18.2%) reported mild pain. Also, most participants reported no downtime following the procedure (90.9%).

### 3.1 Self‐Reported Assessment

**FIGURE 2:**
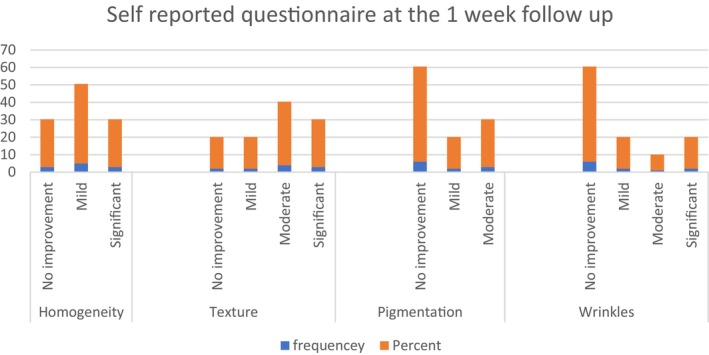
Self‐reported questionnaire at the 1‐week follow‐up. Self‐assessment questionnaire completed by participants 1 week after the procedure. The questionnaire evaluated patient‐reported changes in skin homogeneity, texture, pigmentation, and wrinkles, each rated on a four‐point scale (0 = no improvement, 1 = mild, 2 = moderate, and 3 = significant improvement).

At the 1‐week follow‐up, most patients reported a perceived improvement in skin homogeneity (45% mild and 27% significant improvement), as well as improvement in the skin texture (18.2% mild, 36.4% moderate, and 27.3% significant improvement, Figure 2). On the other hand, most participants reported no—or minimal—improvement in the skin pigmentation (54.4% none, 18.2% mild, and 27.3% moderate improvement, Figure 2). Similarly, 54.4% reported no improvement in wrinkles (18.2% mild, 9.1% moderate, and 18.2% significant improvement, Figure 2).

### 3.2 Dermatologist Baseline Assessment—Interobserver Agreement

For the initial assessment, standardized photos taken before the PRP‐microneedling procedure showed no statistically significant difference in the average scores given by each dermatologist for crow's feet (1.15 ± 1.34, 0.923 ± 1.12, 0.69 ± 0.85, *p* = 0.102, Table 1) and skin texture (0.92 ± 1.61, 1.31 ± 1.11, 1.54 ± 1.45, *p* = 0.214, Table 1). However, there was a statistically significant, though not substantial, disagreement among dermatologists regarding their scores for periorbital pigmentation (2.46 ± 1.13, 1.46 ± 0.10, 2.54 ± 1.20, *p* = 0.003, Table 1) and wrinkles (0.92 ± 1.61, 1.23 ± 1.09, 2.38 ± 1.19, *p* = 0.016, Table 1). The average interclass correlation (ICC) for these variables was high (average ICC ≥ 0.5, Table 2).

**TABLE 1:** Describes the average score given by each dermatologist at the first visit (pretreatment).

At visit 1	Attending 1	Attending 2	Attending 3	*p* *
Crow's feet	1.15 ± 1.34	0.92 ± 1.12	0.69 ± 0.85	0.102
Texture	0.92 ± 1.61	1.31 ± 1.11	1.54 ± 1.45	0.214
Pigmentation	2.46 ± 1.13	1.46 ± 0.10	2.54 ± 1.20	0.003
Wrinkles	0.92 ± 1.614	1.23 ± 1.09	2.38 ± 1.19	0.016

*Note:* Average scores given by each dermatologist at the first visit (pretreatment) across different facial assessment parameters. Scores were provided by three attending dermatologists and are presented as mean ± standard deviation. The parameters assessed include crow's feet, texture, pigmentation, and wrinkles.

* *p* < 0.05 was considered to be significant.

**TABLE 2:** Interclass correlation coefficient (ICC) for the three dermatologists.

	Visit 1		Visit 2		Visit 3	
	Average measure ICC	95% CI	Average measure ICC	95% CI	Average measure ICC	95% CI
Crow's feet	0.915	0.785–0.972	0.930	0.8200–0.978	0.917	0.745–0.980
Texture	0.873	0.684–0.957	0.747	0.368–0.919	0.758	0.286–0.939
Wrinkles	0.666	0.185–0.886	0.691	0.191–0.902	0.521	−0.075–0.861

*Note:* Interclass correlation coefficient (ICC) for the three dermatologists across visits 1, 2, and 3. The ICC values, along with the 95% confidence intervals (CI), are presented for each facial assessment parameter (crow's feet, texture, and wrinkles) to assess the consistency of ratings among the dermatologists.

### 3.3 Dermatologist Assessment—Before and After Procedure

The results in Table 2 display the average scores and standard deviations assigned by three dermatologists during three distinct visits (visits 1, 2, and 3) for four key skin attributes: crow's feet, texture, pigmentation, and wrinkles. While subtle fluctuations in average scores are observed between visits for most attributes, these differences are not deemed statistically significant. Specifically, the *p*‐values for crow's feet, texture, pigmentation, and wrinkles are 0.368, 0.135, 0.553, and 0.582, respectively (see Table 3). These *p*‐values suggest that there is no statistically significant variation in dermatologists' assessments across the three visits for these specific skin characteristics.

**TABLE 3:** Average of the scores given by the three dermatologists at visits 1, 2, and 3.

	Visit 1	Visit 2	Visit 3	*p* *
Crow's feet	0.67 ± 0.82	0.85 ± 1.02	0.81 ± 1.09	0.368
Texture	0.96 ± 0.99	1.33 ± 0.8	1.15 ± 1.03	0.135
Pigmentation	2.18 ± 0.91	2.52 ± 0.80	2.22 ± 0.96	0.553
Wrinkles	1.41 ± 0.91	1.48 ± 0.87	1.55 ± 0.93	0.582

*Note:* Average scores given by the three dermatologists at visits 1, 2, and 3 across different facial assessment parameters. The parameters evaluated include crow's feet, texture, pigmentation, and wrinkles. Scores are presented as mean ± standard deviation.

* *p* < 0.05 was considered to be significant.

## 4 Discussion

Microneedling is the technique that involves the use of multiple needles at controlled depths to create thousands of microwounds in the dermis that stimulate the dermal wound healing cascade regenerative mechanisms to improve the skin quality [1]. It has been shown to increase the production of platelet‐derived growth factor, fibroblast growth factor, and transforming growth factor (TGF α and β), which contributes to fibroblast proliferation and migration, neovascularization, and neocollagenesis [1].

PRP is an autologous solution rich in multiple growth factors such as platelet‐derived growth factor (PDGF), TGF, vascular endothelial growth factor (VEGF), and epithelial growth factor [3]. Injections of PRP have been utilized in multiple disciplines of medicine, including various skin treatments [3]. PRP injections are thought to contribute to skin rejuvenation through fibroblast activation, collagen synthesis, and by providing other matrix components and various growth factors [3].

The concept of using activated PRP to enhance the regenerative cascade triggered by various ablative skin treatments has been studied. In fact, PRP has been employed as an adjuvant therapy to improve the results of cosmetic lasers, potentially boosting the tissue remodeling process after the controlled damage caused by lasers [10]. Similarly, PRP combined with microneedling has also been shown to be effective in treating acne scars and improving skin quality [10].

Our study aims to objectively assess the potential benefits of PRP combined with microneedling for skin rejuvenation, focusing on periorbital wrinkles, crow's feet, pigmentation, and skin texture. There are existing studies examining their individual effects [3, 11, 12, 13, 14, 15, 16, 17] and very few looking at their combined effects [18, 19, 20].

Many of these previous studies lack randomization and blinding and have relied on subjective assessment tools such as patient satisfaction questionnaires and treating physician self‐assessments, which can be influenced by various biases and confounding factors. Our paper aimed to utilize objective methods of assessment including randomization and blinding methods and standardized photography methods that all minimize many of the confounding variables.

In our study, most patients self‐reported a perceived improvement in skin homogeneity, texture, and pigmentation, with nearly half also noting an improvement in wrinkles. However, these self‐assessments were contrasted by the evaluations of independent dermatologists, who found no significant differences in any of the parameters measured. Our findings are consistent with the only randomized controlled trial looking into the combination of PRP and microneedling. Alam et al. [11] compared PRP injections without microneedling to saline injections using a split‐face design. In that study, participants observed an improvement in skin texture after a single PRP treatment, but two independent blinded dermatologists found no significant differences between PRP and saline in photoaging scores, including assessments of fine lines, mottled pigmentation, roughness, and sallowness.

It has been hypothesized that doing more than a single treatment session might be more beneficial. The only randomized controlled trial by Merati et al. evaluated four consecutive sessions of microneedling, with and without the addition of a novel human recombinant growth factor regenerative complex (PolyGF), found improvement only in skin texture when assessed using objective imaging analysis (Canfield VISIA Complexion Analysis System, Fairfield, New Jersey). However, no significant improvement was observed in other measured parameters, including age spots, evenness of skin tone, crow's feet, nasolabial folds, and frown lines [20].

Although there are no other randomized controlled trials looking into the combination treatment, many studies investigated their individual effects did not find substantial results. A placebo‐controlled split‐face study of 4 weekly injections of PRP without microneedling in the crow's feet and preauricular areas found no significant differences in wrinkle reduction between the two sides at 3 months posttreatment. Nevertheless, 75% of the patients were satisfied with their results [17]. Additionally, another study involving a single PRP injections without microneedling in crow's feet and infraorbital area showed subjective improvements in infraorbital color homogeneity, but no significant changes in melanin content, stratum corneum hydration, wrinkle volume, and visibility index [16].

The discrepancies in results between studies may be attributed to various factors, including differences in study design, treatment protocols, and assessment methods, as well as the presence of multiple biases. Notably, the lack of standardized PRP preparation protocols across studies, along with variations in the number of treatment sessions, likely contributes to these inconsistencies. The preparation and concentration of PRP can significantly influence its efficacy, leading to varying outcomes [21]. Additionally, it appears that a greater number of treatment sessions may yield better results in skin quality, as studies showing positive improvements typically involved at least three sessions of PRP or microneedling [12, 14, 19, 20].

It is noteworthy that most studies relying on patient self‐assessments, including our own, as well as evaluations by the treating physician, consistently reported positive improvements. In contrast, studies utilizing blinded independent observers revealed more variability in results and generally lower levels of improvement. This discrepancy may be attributed to confirmation and expectation biases affecting both patients and treating physicians. An interesting study by Banihashemi et al. showcased these biases clearly. The study looked into the effects of two PRP injections, administered 3 months apart without microneedling, on improvements in periorbital dark circles, wrinkles, nasolabial folds, and skin rigidity as assessed by patients, the treating physician and a second blinded physician. Patients reported greater improvements across all parameters compared to the treating physician, who in turn rated the improvements higher than the second blinded dermatologist [13]. In our study, we employed three independent dermatologists who were blinded to the sequence of photos, which were randomized across the entire pool of participants. This approach effectively minimizes any potential expectation or confirmation biases.

On a histologic level, several studies have demonstrated that three consecutive monthly treatments with PRP can lead to histometric improvements in skin quality, including enhanced cutometer measurements, improved skin barrier function, and increased capacitance [12, 14]. Furthermore, a study involving six consecutive biweekly treatments with combined PRP and microneedling showed histopathological improvements in dermal structures, as evidenced by skin punch biopsies [19]. This study noted a significant increase in epidermal thickness, the organization of collagen bundles, and a marked reduction in abnormal elastic fibers [19]. We believe that while histologic and histometric improvements may be subtle and more noticeable to patients, these changes might not be pronounced enough to be detected by independent expert observers.

Additionally, our study adhered to strict guidelines for standardizing lighting and photography. Variations in lighting angles can significantly alter the perception of facial features in 2D photographs, a factor that must be carefully controlled [22]. To address this, all photographs in our study were taken under consistent lighting conditions, using the same photography lens and settings, and with head mounts to account for positional variables. Our study is limited by the small number of participants, the lack of a control group, and the single treatment session.

In conclusion, despite a plethora of studies on the aesthetic use of PRP and microneedling, there is a lot of variability in treatment and assessment methods as well as results. Although existing studies demonstrate some histologic and objective skin improvements, our findings suggest that a single session of combined PRP and microneedling does not produce significant visible changes, particularly in areas like the eyelids affected by dark circles and other periorbital skin concerns. Future research should include standardized objective assessments, larger sample sizes, more diverse populations, and extended treatment durations to better evaluate the potential benefits of combining PRP and microneedling.

## Author Contributions

S.A.H. conceptualized and designed the study, contributed to data collection, analysis, and interpretation, and drafted the manuscript. D.S.S. contributed to data collection, analysis, and manuscript revision. M.K. was involved in the interpretation of data and critically reviewed the manuscript for important intellectual content. J.A.R. contributed to the study design and manuscript revision. R.M.A. supervised the study, contributed to data interpretation, and provided final approval of the version to be published.

## Ethics Statement

This study was approved by the institutional review board (IRB), BIO‐2017‐0427.

## Conflicts of Interest

The authors declare no conflicts of interest.

## Data Availability

The data that support the findings of this study are available on request from the corresponding author. The data are not publicly available due to privacy or ethical restrictions.
